# Long-term sequel of posterolateral rotatory instability of the elbow: a case report

**DOI:** 10.1186/1749-799X-5-5

**Published:** 2010-01-27

**Authors:** Chun-Ying Cheng

**Affiliations:** 1 Department of Orthopaedic Surgery, Chang Gung Memorial Hospital, Chang Gung University, Taoyuan, Taiwan

## Abstract

The natural course of untreated posterior lateral rotatory instability of the elbow is unclear. A case of elbow arthrosis with progressing deformity and flexion contracture after an episode of elbow dislocation about 20 years ago presented the possibility the long term outcome of untreated posterior lateral rotatory instability of the elbow.

## Introduction

The lateral collateral ligament complex of the elbow is the main stabilized of posterolateral rotatory instability and was described by O'Driscoll at 1991[1]. Posterolateral rotatory instability of the elbow results from insufficiency of the lateral ligamentous and muscular support of the elbow, which allows the radial head and proximal ulna to subluxate away from the humeral capitellum and trochlea when axially loaded in supination [2]. The long term outcome of unrecognized posterior lateral rotatory instability of the elbow is unclear and rarely reported. The author described a case of progressing deformed elbow with flexion contracture after an episode of elbow dislocation about 20 years ago with the symptom of tardy ulna nerve palsy for 4 months; the ulnar nerve symptom and elbow function was improved after a surgical repair of the lateral collateral ligament complex and anterior transposition of the ulnar nerve. This case of elbow arthrosis presented the possibility of the nature course of posterior lateral rotatory instability of the elbow.

## Case presentation

A 46-year-old, right-hand-dominant male presented with left ring and little fingers numbness and hand weakness that had been aggravated over the previous 4 months. He had chronic pain and progressive deformity of lateral elbow, and lost extension after one episode of elbow dislocation about 20 years ago. He was transferred to our office for further assessment with above symptoms. Tracing back his trauma history revealed that he noted a daily sensation of painful slip in and out on the lateral elbow joint after a dislocation underwent a closed reduction by a bonesetter. His elbow symptom didn't improve or got a diagnosis after visiting three orthopedic surgeons for the first 6 months. Although his elbow symptom was persisting but he was tolerable at eating, dressing, carrying or pulling of daily activity or working ability except lifting or push-up and he didn't visiting any physician for further help since then until this new symptom of hand numbness occurred.

**Figure 1:**
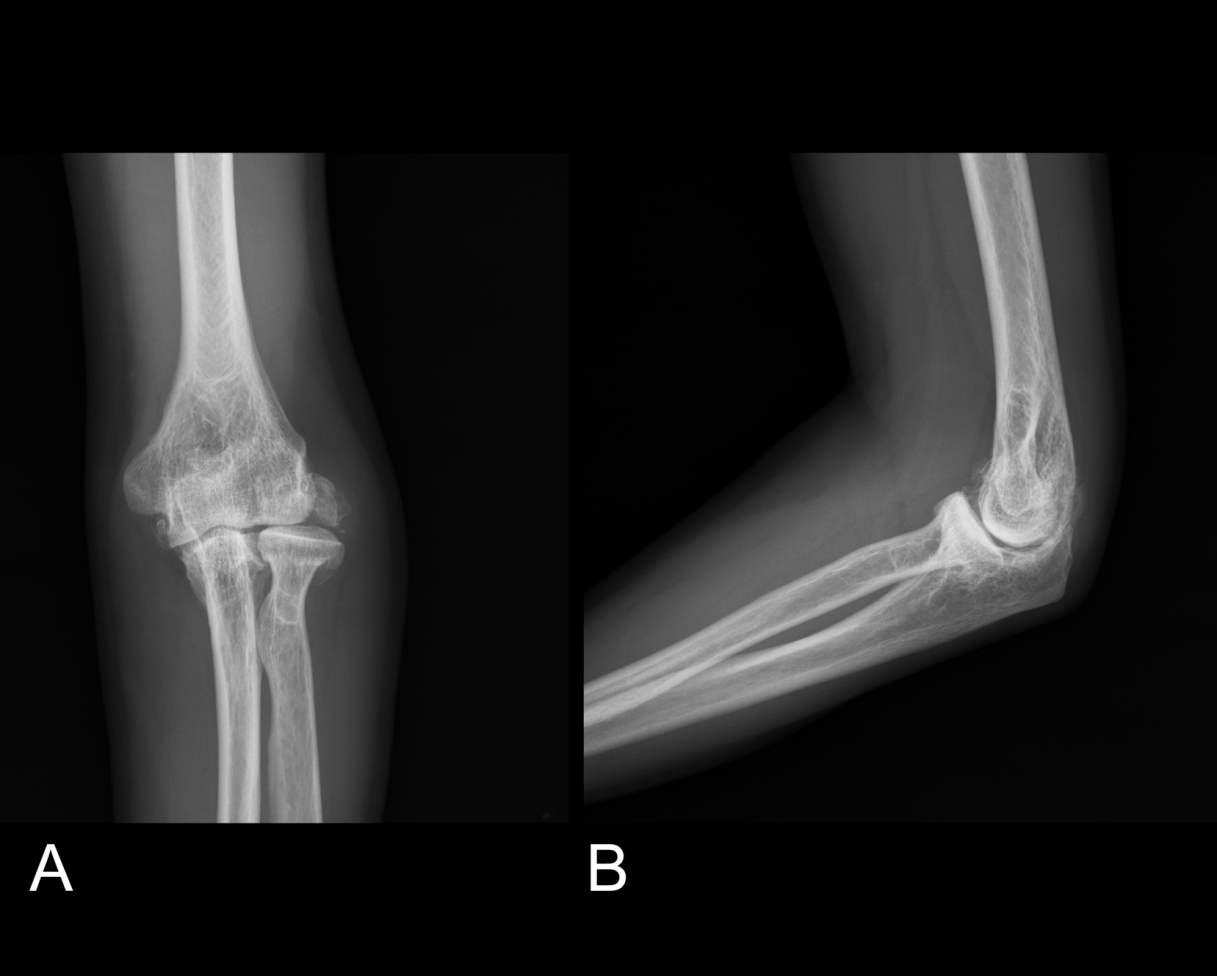
**(A) Posteroanterior (B) lateral radiographs of elbow showed degeneration of ulnohumerus and radiohumerus joint with radial head deformity and subluxcation and avulsed bone around the lateral epicondyle**.

Physical examination revealed the elbow with flexion arc from 20° to 120° and full forearm rotation compared with contra lateral side, and grip strength 105 lb (125 lb on the right side). Palpation revealed the deformed elbow with prominent radial head not lateral epicondyle on the lateral of the elbow. The result of neurologic examination was abnormal including paresthesias in the ulnar half of ring finger and little finger and dorsal ulnar wrist with positive Tinnel sign and nerve compression test of the ulnar nerve at elbow, little finger abduction weakness but without claw hand deformity. Plain radiographs showed arthrosis of the elbow joint with the radiohumeral joint more sever than ulnohumeral joint, radial head deformity including lost normal concave shape and hypertrophic marginal osteophyte with lateral subluxation and some chip bone or ectopic bone over lateral epicondyle (Fig. 1). Patient was arranged to receive operation with the surgical plan to decompress the ulnar nerve by anterior transposition of the nerve and evaluate the elbow joint stability under anesthesia.

**Figure 2:**
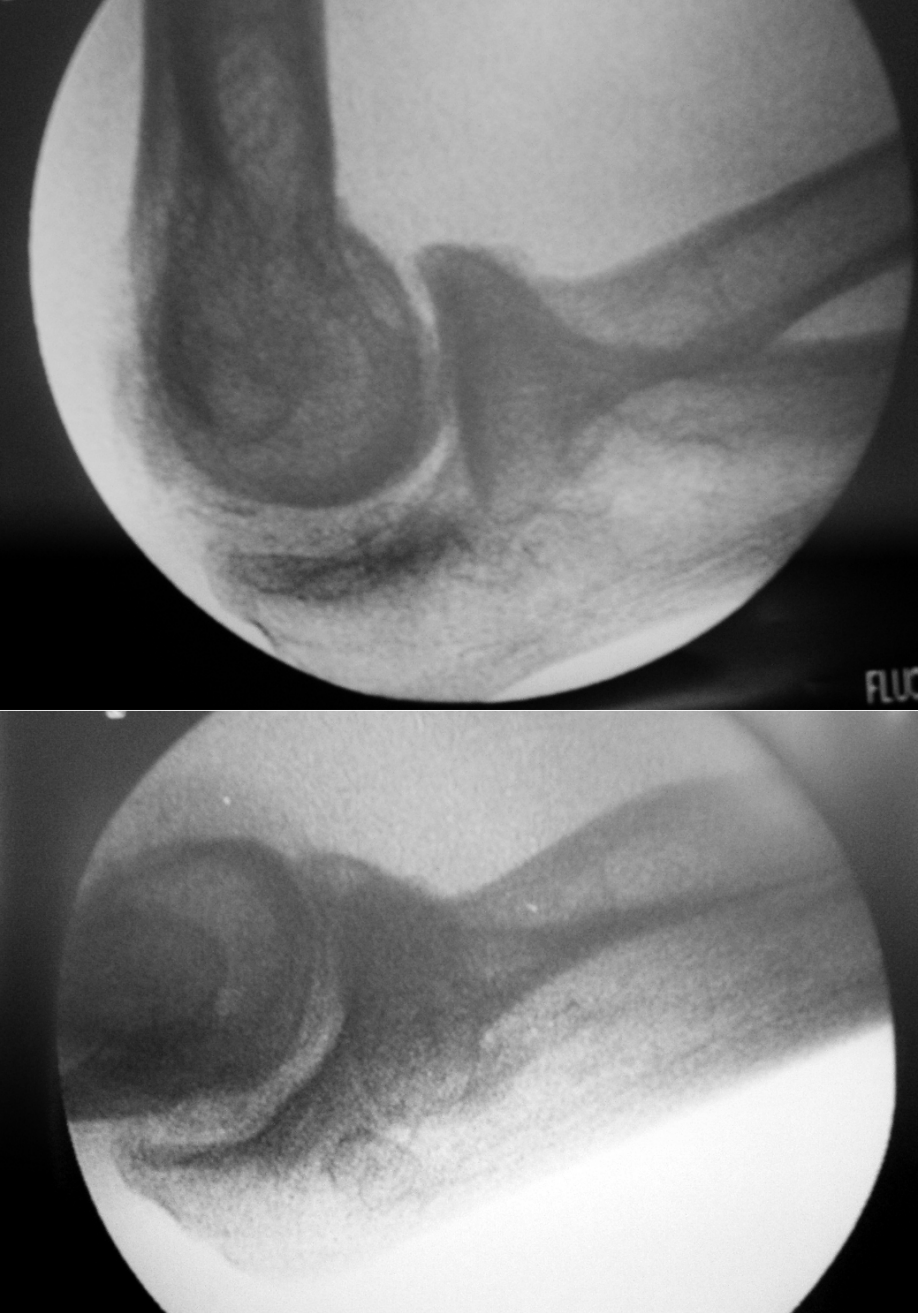
**Fluroscan of elbow without and with lateral stress (provocative stress applied) showed subluxed radial head posterior to the midline of the capitellum**.

After general anesthesia, the lateral pivot shift test by O'Driscoll's method [1] with the patient's arm overhead was positive and the elbow stress test at fluoroscan revealed negative valgus and varus stress test and positive lateral stress test [2], which the radiograph is taken with provocative stress applied during the lateral pivot shift test (Fig. 2). The operation was performed with the patient positioned supinely and supported by a hand table. The elbow was approached with two separate lateral and medial incision. The traction neuropathy of ulnar nerve at cubital tunnel was noted and intact medial collateral ligament was identified after subcutaneously anterior transposition of the ulnar nerve. The lateral structure was exposure through the Kocher interval and an avulsed bone fragment of lateral collateral ligament complex including common extensor from lateral epicondyle was noted, the radial head was found to translate posterior by provocative test stress at 30° of flexion and the annular ligament was found to be intact. The lateral collateral ligament complex was repaired with a bone anchor with No.2 polyester braided non-absorbable suture, which in a running locked fashion at origin of tendon and ligament [2] and augmented with a bone screw to fix the avulsed fragment. Postoperatively, the elbow was protected by a hinged brace with the forearm in a neutral position for 4 to 6 weeks and the flexion angle of the brace was allowed to step decreased 10° per week. Progressing loading and strengthening are permitted for the late of 2 to 6 months.

**Figure 3:**
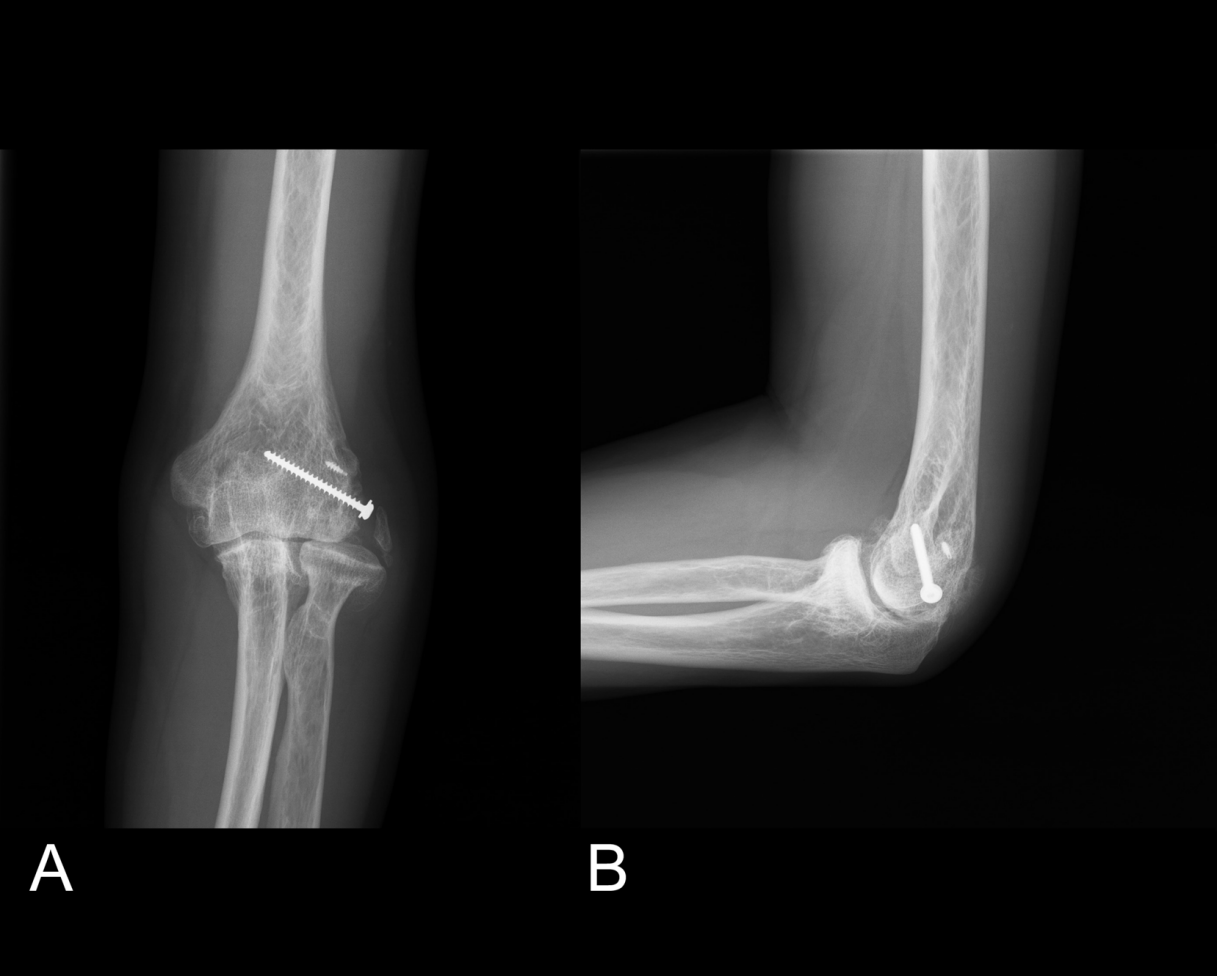
**(A)Posteroanterior (B) lateral radiographs of elbow 2 years after operation showed anchor suture and bone screw at lateral epicondyle and incompletely reattached avulsed bone**.

At 24 months after surgery, the patient was satisfied with the procedure; the symptom of ulnar nerve was recovering and he felt that his elbow was more comfortable and stable at daily activities except lifting. Examination revealed motion from 10° of extension to 130° of flexion, 75° of pronation and 80° supination, and no signs instability and grip strength increased to left 115 lb (126 lb on the right). Post-operative plain radiographs showed the deformed radial head still subluxation at anterior-posterior view but no progressing arthrosis of the elbow joint(Fig. 3).

## Discussion

Neglected or under-diagnosis the posterolateral rotatory instability of the elbow is possible because plain radiographs are commonly nondiagnosised. The symptoms of this condition including pain, instability or mechanical snapping or popping are subtle and relevant. The clinical assessment of subluxation and reduction sometimes by provocative test is hampered by patient apprehension and guarding or only detected under anesthesia. Most orthopedics surgeon didn't understand the existence of posterior lateral rotatory instability before the Dr. O'Driscoll's description at 1991 [1].

The patient presented the symptom and sign of loss of extension, degenerative changes in the joint, ectopic calcification or neurological changes are common residual sign and symptom following elbow dislocation [3,4]. The patient's symptom of radial head subluxation and lost concave deformity of radial head without symptom of forearm rotation and the sign of plain radiographs showed arthrosis of the elbow joint with the radiohumeral joint more sever than ulnohumeral joint are different from the consequence of simple elbow dislocation or radial head dislocation. The diagnosis of posterolateal rotatory instability in this case is undoubted because there is positive lateral pivot shift test and lateral stress test of fluoroscan under anesthesia and identified avulsed fragment of lateral collateral ligament complex during operation.

The cause of joint degeneration may be multiple factors, but the relation of joint instability and joint degeneration is interesting and deserving to be concern. The relation of scapholunate ligament injury or scapholunate dissociation (instability) in the wrist with scapholunate advanced collapsed degeneration is well known; we need more clinical studies of posterolatreal rotatory instability of the elbow and biomechanical investigations of the pivot-shift test of lateral collateral ligament complex to establish this relationship and understanding the natural course of posterolateral rotatoy istability of the elbow. The radiographic findings of this case with elbow arthrosis more severs on the radiohumeral joint than ulnohumeral joint and the radial head hypertrophic deformity and subluxation may be to characterize a neglected ligament injury with rotatory instability.

## Consent

Written informed consent was obtained from the patient for publication of this case report and accompanying images. A copy of the written consent is available for review by the Editor-in-Chief of this journal.

## Competing interests

The author declares that they have no competing interests.
